# Anaesthetic Tricaine Acts Preferentially on Neural Voltage-Gated Sodium Channels and Fails to Block Directly Evoked Muscle Contraction

**DOI:** 10.1371/journal.pone.0103751

**Published:** 2014-08-04

**Authors:** Seetharamaiah Attili, Simon M. Hughes

**Affiliations:** Randall Division of Cell and Molecular Biophysics, Guy's Campus, King's College London, London, United Kingdom; Dalhousie University, Canada

## Abstract

Movements in animals arise through concerted action of neurons and skeletal muscle. General anaesthetics prevent movement and cause loss of consciousness by blocking neural function. Anaesthetics of the amino amide-class are thought to act by blockade of voltage-gated sodium channels. In fish, the commonly used anaesthetic tricaine methanesulphonate, also known as 3-aminobenzoic acid ethyl ester, metacaine or MS-222, causes loss of consciousness. However, its role in blocking action potentials in distinct excitable cells is unclear, raising the possibility that tricaine could act as a neuromuscular blocking agent directly causing paralysis. Here we use evoked electrical stimulation to show that tricaine efficiently blocks neural action potentials, but does not prevent directly evoked muscle contraction. Nifedipine-sensitive L-type Ca_v_ channels affecting movement are also primarily neural, suggesting that muscle Na_v_ channels are relatively insensitive to tricaine. These findings show that tricaine used at standard concentrations in zebrafish larvae does not paralyse muscle, thereby diminishing concern that a direct action on muscle could mask a lack of general anaesthesia.

## Introduction

In the 18^th^ century, Luigi Galvani laid the foundations of biophysics by discovering that electrical stimuli can trigger muscular contraction, transmit to muscles via nerves and are also generated by animals themselves [1]. Since that time, neurons and skeletal muscle have been shown to be electrically excitable; electrical action potentials in neurons trigger further action potentials in muscle fibres that lead to contraction. Voltage gated ion channels are key to the generation of action potentials (reviewed in [2]). Neuron and muscle have subtly different electrical properties, each employing distinct voltage-gated channels. Normally, an action potential propagates rapidly along the neural axon, stimulating the release of the neurotransmitter, which in turn elicits an action potential in the muscle fibre plasma membrane. This second action potential propagates via the transverse-tubule system deep into the fibre, where the dihyropyridine receptor (DHPR) acts as a voltage sensor that causes release of Ca^++^ from sarcoplasmic reticulum into the cytoplasm. Increased cytoplasmic Ca^++^ concentration promotes actomyosin contractility [3], [4]. Following Galvani, muscle contraction can be directly activated by external electric stimulation that triggers action, controlling not only immediate contraction events but also longer term muscle gene expression [5]–[8].

Excitability arises from the sequential opening and closing of voltage-gated Na^+^, Ca^++^ and K^+^ channels [9], [10]. In adult skeletal muscle, voltage-gated Na^+^ (Na_v_) channels initiate the action potential, rapidly activate and inactivate in a few milliseconds and have been widely studied [2], [11]. In embryonic tissues, however, voltage–gated Ca^++^ (Ca_v_) channels can make a significant contribution to the action potential [12]–[14]. Additionally, L-type voltage-gated Ca_V_1.1 channels play an essential role in calcium release in skeletal muscle through their role as components of the DHPR, which provides a voltage-sensitive mechanical link that triggers release of Ca^++^ from the sarcoplasmic reticulum [12], [15]. Drugs that target voltage-gated channels have proved invaluable in the clinic, but often have side effects due to their ability to bind more than one channel type [16]. Local anaesthetics, for example, inhibit the generation of neural action potentials by blocking the flow of ions through voltage-gated ion channels [17]–[19]. However, compounds such as lidocaine and bupivacaine can have side effects through, for example, cardiac depression.

The larvae of the zebrafish *Danio rerio* offer many advantages for drug screening and investigating the relationship between ion channel expression and nerve and muscle function *in vivo* [20], [21]. The practical use of zebrafish often involves, however, the use of tricaine, which is a rapidly reversible general anaesthetic that supresses movement and thus permits visualisation and manipulation of the organism. Tricaine methanesulfonate (also known as 3-aminobenzoic acid ethyl ester or MS-222) is a preferred drug for aquatic anaesthesia in Norway [22] and in zebrafish experimentation by the U.K. Home Office [23] and is the only drug approved for use in fish by U.S. Food and Drug Administration (www.accessdata.fda.gov/scripts/animaldrugsatfda/). Previous reports have shown a suppressive effect of tricaine on zebrafish peripheral and central nervous system [24]-[26], but also adverse effects on physiology (summarised in [22]). Tricaine, at the normal concentrations used, has little effect on embryonic cardiac contractility, but the heart becomes more sensitive as animals mature [27]. The molecular and cellular targets of tricaine in zebrafish are not fully characterised. This is particularly important because the use of tricaine has recently been questioned on the grounds that fish sense and choose to avoid tricaine [23], [28]. It would be of great concern if tricaine were to act directly to paralyse skeletal muscle, thereby preventing movement, as this might mask a lack of general anaesthesia.

Local anaesthetics of the amino amide class, to which tricaine belongs, are known to inhibit certain mammalian Na_v_ channels e.g. [29]. Such channels are generally composed of alpha and beta subunits and are primarily defined by their large alpha subunit, which contains the voltage-sensing domain [2]. The zebrafish sodium channel (*scn*) gene family consists of four sets of duplicated alpha subunit genes. *scn1a* and *scn1Lab* form the Na_v_1.1 channels expressed in cardiac muscle [30]. *scn4aa* and *scn4ab* contribute to Na_v_1.4 channels and are expressed in skeletal muscle [30]–[32]. The expression of *scn12aa* and *scn12ab* (putatively forming Na_v_1.5 channels) is transient in zebrafish muscle [31], but in mouse *Scn5a* is expressed in neonatal skeletal muscle and limbic system and in adult muscle after denervation [33]. *scn8aa* and *scn8ab* (Na_v_1.6 channels) are expressed in brain eye, spinal cord, retina, and peripheral nervous system [30], [31], [34]. The zebrafish Na_v_ channels targeted by tricaine are unclear.

Whether amino amide anaesthetics also inhibit zebrafish Ca_v_ channels is unknown, but such effects have been reported in other species [35]–[38]. Ca_v_ channels have similarities to Na_v_ channels, in that their alpha subunits have clear structural and functional similarities [39]. Zebrafish somites make the first skeletal muscle and express L-type voltage gated calcium channels *cacna1sa* (CaV1.1)[40]. In contrast, the P/Q-type *cacna1a* is expressed in brain, neuronal cells and heart [40]. Thus, the channels and cell types inhibited by tricaine are not fully characterised.

We set out to investigate the tissue target(s) of tricaine in the zebrafish, particularly in muscle. We report the impact of tricaine on zebrafish skeletal muscle at early developmental stages. We also studied the impact of nifedipine, an L-type Ca^++^ channel antagonist. Our findings show that, at the normal concentrations used for anaesthesia, tricaine does not prevent muscle contraction or interfere with excitation-contraction coupling.

## Materials and Methods

### Animals and Reagents

Danio rerio, either Tübingen wild type or *chrnd^sb13^* (here called ‘*fixe*’) mutants [41] maintained on Tübingen background, were reared at 28.5°C according the standard procedures [42]. All experiments were performed at age of 3-3.5 days post-fertilization (dpf). Stock aliquots (2 ml) of 16 mM Tricaine (3-aminobenzoic acid ethyl ester methanesulphonate) in double distilled water at pH 7 were stored at -20°C. Agarose (Sigma Life Sciences) 2% in fish water was prepared and stored in fridge at 4°C. Aliquots (10 µl) of Nifedipine (Sigma-Aldrich) in dimethylsulfoxide (DMSO) at stock concentration of 100 mM were stored at −20°C. Tricaine and Nifedipine were thawed at room temperature each day of experiment prior to dilution.

### Electrical Stimulation

**Figure 1:**
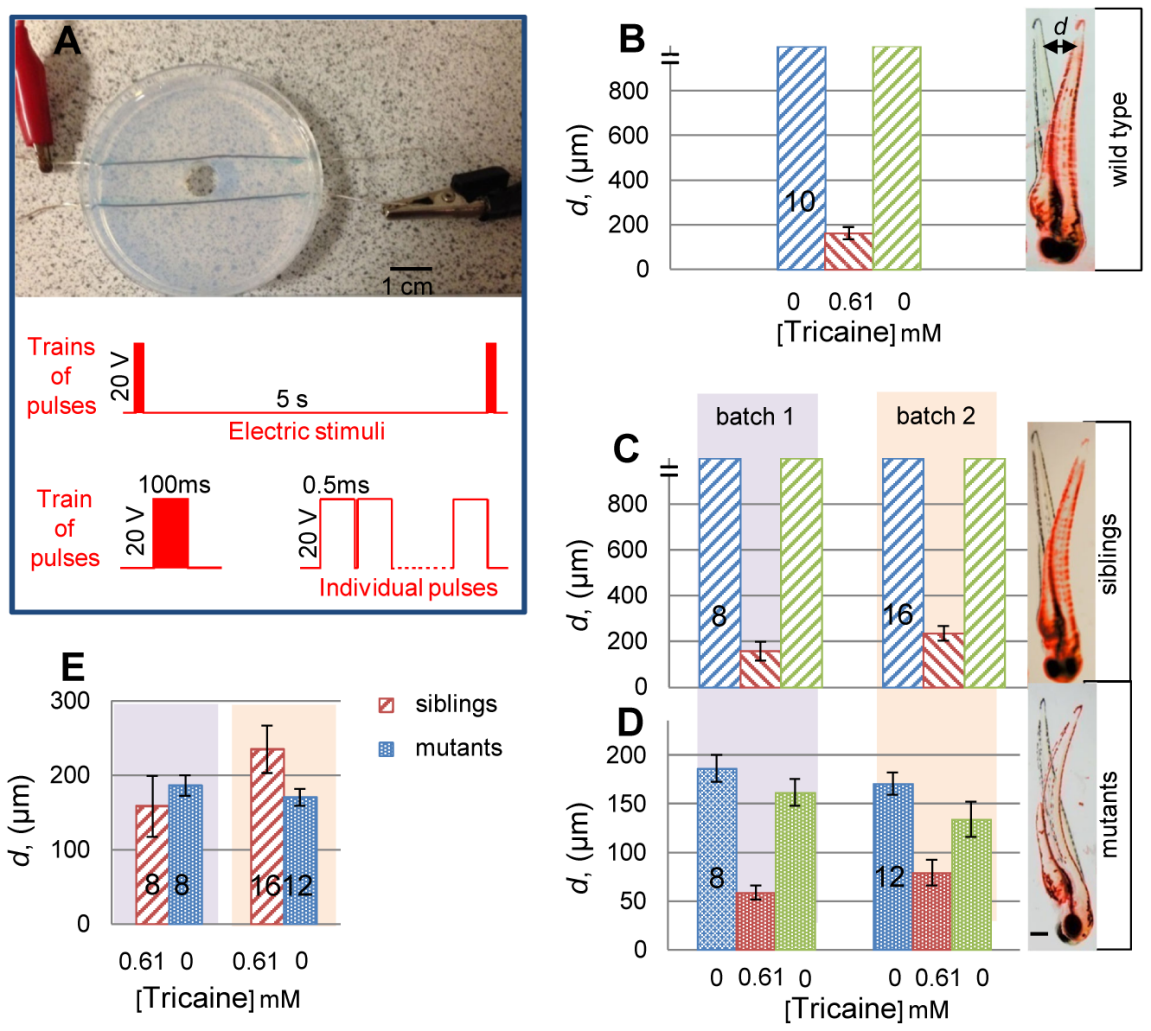
Tricaine fails to block evoked muscle contractility. **A.** The electrical stimulation set-up (ESS). Fish are placed within the central well aligned, when spontaneous motility permits, with their anteroposterior axis perpendicular to the electrodes. Schematic of the electric stimulus regime is shown below. **B-D**. Tail displacement (*d*) in embryos from wild type (B) or a *fixe* heterozygote incross sorted into motile siblings (C; *chrnd^+^* ^/*?*^ 97/132 = 73.5%) and immotile mutants (D; *chrnd^sb13^* ^/*sb13*^ 35/132 = 26.5%). Movement was quantified from videos as measured displacement (*d*) of tail as shown (B-D, right panels) in superimposed stills of single fish before stimulation (black image) and at maximum displacement (false-coloured red image). Unanaesthetized motile fish move extensively, generally out of the field of view (indicated by break in *Y*-axis). Fish were measured before tricaine exposure, after exposure to 0.61 mM tricaine for 30 min and after ≤30 minutes of tricaine washout into fish water. Note altered *Y*-scale in D compared to B,C. **E.** Comparison of mutants with tricaine-treated individuals reveals a striking similarity in displacement. Error bars are SEM. Numbers of embryos are shown on bars. Scale bars  = 0.1 mm.

Fish larvae were subjected electrical stimulation at room temperature using a custom-built electric stimulation set-up (ESS) (Fig. 1A). To minimize damage to the fish, the ESS consists of two silver electrodes (0.5 mm ∅, 99.9%, Arcos Organics, New Jersey, U.S.A.) arrayed parallel and 10 mm apart passing through the drilled holes of a 6-well tissue culture plate or a 60 mm ∅ petri dish. Chambers with electrodes were filled with 2% agarose gel, such that the silver electrodes are completely within the gel. A circular well of volume 0.5 mL was created by removing the agarose in the centre of the chamber between the two electrodes. Fish are placed carefully into the well with desired orientation. Care was taken not to expose the electrodes to the fish swimming in the well. The electrodes are connected to a stimulator (Grass S-88, Grass Instruments, U.S.A) that generated square wave pulse trains of indicated lengths. Polarity of electrodes was reversed between pulse trains to avoid electrolysis. A range of stimulation regimes was tested, with the aim of attaining a brief twitch response, and all experiments shown used a train of 200 20 V pulses, with 0.5 ms pulse duration (100 ms total train duration) once every 5 seconds (Fig. 1A).

### Drug treatments

For wash-out, fish were later transferred to a dish containing fresh fish water and were left there for over 30 minutes. Care was taken to exchange the fish water five times to remove residual tricaine.

### Data capture and analysis

Observations were made using a dissecting microscope (Leica MZ 16F, Germany). All videos were recorded using Olympus (DP70) camera with DP Controller software at a frame rate of 60 frames per second in total time of 25 seconds.

Fish tail displacement “*d*” was measured at the level of the last somite by superimposing images from video. Two images, before and after the onset of stimulation, were overlaid using Adobe Photoshop by false-colouring one image red and converting it to transparent mode.

### Ethics Statement

Fish were bred and experiments performed under licences from the UK Home Office and conform to all institutional, national and EU guidelines.

## Results

The performance of ESS was tested at a range of voltage and pulse train parameters to define a stimulation regime that was not toxic to the fish yet triggered motility. The response of fish to the external stimulus was immediate oscillatory swimming movement with the appearance of an escape response (Movie S1). Escape responses are under neural control either at spinal or higher levels [43]. Evoked movement was quantified by measuring tail tip displacement at the level of the last somite upon stimulation and was >1 mm in sentient fish (Fig. 1B,C). To determine whether electrical stimulation also acts directly on muscle cells, *fixe* mutant larvae, which lack functional acetylcholine receptors [41], were stimulated and gave a single strong contraction to each pulse train that subsequently relaxed (Movie S1). Maximal tail displacement was about 0.17 mm (Fig. 1D). Thus, electric stimulation evokes muscle fibre contraction directly.

Amino amide anaesthetics are known to hinder the generation of action potentials by blocking diffusion of ions through voltage-gated channels within excitable cells, thereby inhibiting the movement [18], [44]. Adding tricaine to wild type or non-mutant *fixe* sibling larvae at the concentration widely used for anaesthetizing zebrafish (0.61 mM; 160 mg/L)[42] completely inhibited movement in response to touch, water flow or vibration (data not shown). However, tricaine did not block motility evoked by electrical stimulation (Movie S1). Strikingly, the contraction/relaxation timecourse and maximal displacement in anaesthetised fish was indistinguishable from that evoked in *fixe* mutants (Fig. 1E). The action of tricaine was fully reversible, as fish regained normal movement after washout of the anaesthetic (Fig. 1B,C). We conclude that tricaine anaesthesia does not prevent action potentials, excitation-contraction coupling or actomyosin contractility in zebrafish muscle fibres. We observed no difference in measured movements between wild type and non-mutant *fixe* siblings; we continued our experiments with *fixe* siblings.

**Figure 2:**
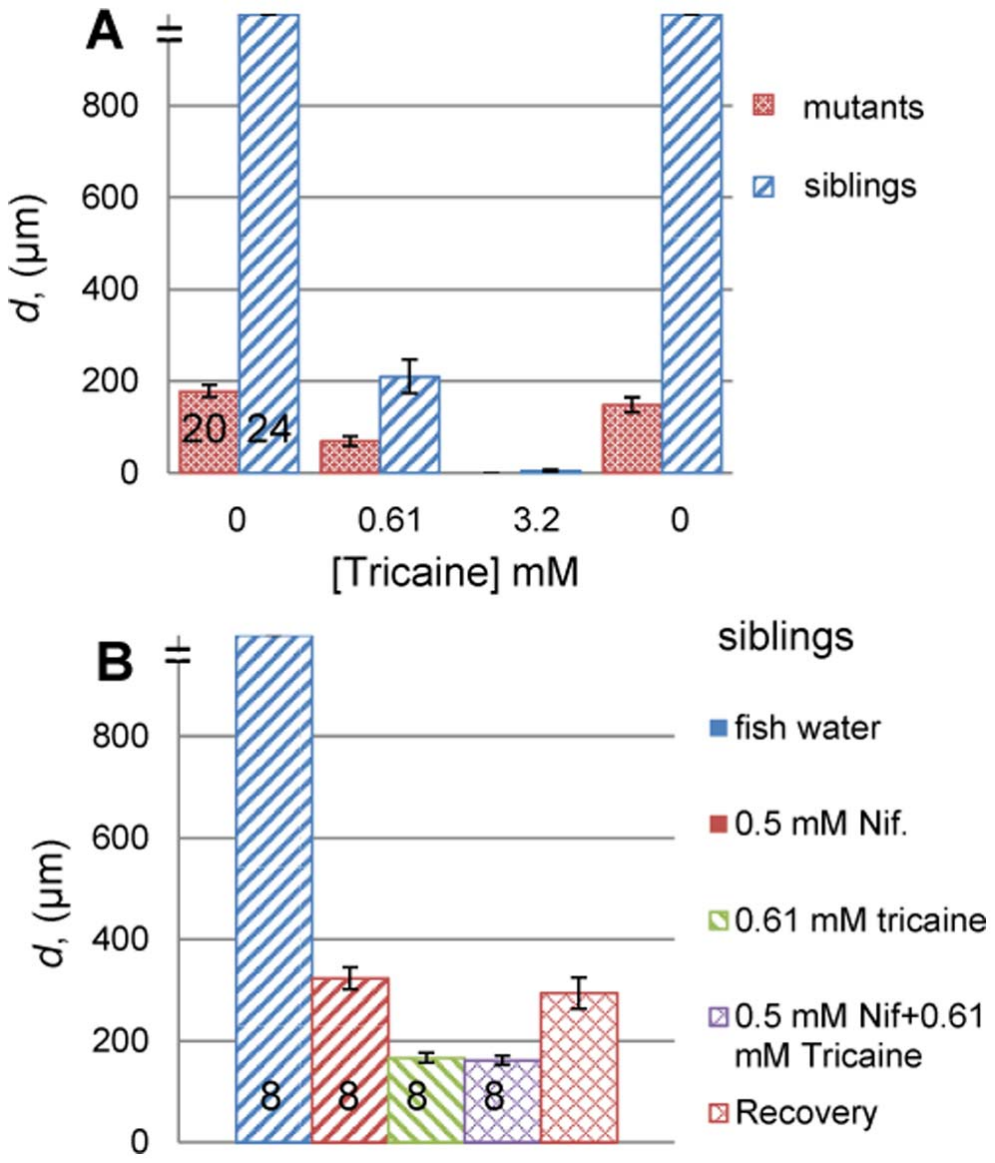
Tricaine blocks muscle contraction at high concentrations. **A.** Comparison of tail displacement (*d*) as a function of successively increasing tricaine concentration in *fixe* mutants and their siblings. In both cases, full movement is regained upon tricaine wash-out to fish water. **B.** Tail displacement in response to L-type Ca_v_ channel blocker Nifedipine alone, tricaine alone, or both, and after wash-out of both drugs (right hand bar). Error bars are SEM. Numbers of embryos are shown on bars.

Given that both tricaine and loss of acetylcholine receptors had the same effect of blocking the neurally-induced escape response but not electric stimulation-evoked motility, we predicted no effect of adding tricaine to *fixe* mutants. Strikingly, however, we consistently observed a reduction in evoked motility (Movie S1 and Fig. 1D). Again, the effect was reversible upon washout of tricaine. These observations confirmed that muscle fibres are resistant to the suppression of action potentials by tricaine, but further suggested that tricaine does act weakly on muscle fibre ion channels, at least those present in immotile *fixe* mutants. To test this hypothesis, higher doses of tricaine were applied to wild type or *fixe* sibling larvae. Motility gradually declined at successively higher doses until it was essentially inhibited by 3.2 mM tricaine (Fig. 2A and data not shown). In high dose tricaine, *fixe* mutants did not move detectably in response to stimulation, whereas their siblings still showed very slight twitches that caused negligible tail displacement (Movie S1). Thus, tricaine can directly inhibit striated muscle contraction, but functionally denervated muscle appears more sensitive to tricaine than muscle in unmanipulated larvae.

Why is muscle resistant to doses of tricaine that act as a general anaesthetic? One hypothesis is that the voltage-gated channels in skeletal muscle are distinct from those inhibited by tricaine in the nervous system. As Na_v_ channels are known to be major targets of tricaine in neurons [18], we asked whether action potentials in larval muscle are Ca^++^-based and thus have reduced sensitivity to tricaine. Nifedipine is a calcium channel blocker used to test the role of Ca_v_ channels in generation of action potential [45]. Application of Nifedipine (0.1 mM) reduced the movement of unanaesthetised fish (data not shown). At higher concentrations (0.5 mM) nifedipine reduced movement significantly but less effectively than tricaine (Fig. 2B). Fish incubated in both 0.5 mM Nifedipine and 0.61 mM tricaine for 30 minutes showed no significant difference from fish treated with tricaine alone (Fig. 2B). When the drugs were washed out with fish water, movement returned to the level elicited by nifedipine alone, consistent with the known irreversible action of nifedipine [46], [47]. Nifedipine did not reduce motility in *fixe* mutants (data not shown). These results suggest that nifedipine-sensitive L-type Ca_v_ channels are functional in neurons of the motor pathway, but do not significantly contribute within the muscle itself.

## Discussion

There are four major results from the current study. First, a simple and custom built electric stimulation set-up shows that larval zebrafish muscle contraction can be directly evoked using external electric stimuli. Second, that tricaine anaesthesia at normal doses prevents movement by effectively inhibiting neural but not skeletal muscle action potentials. Thirdly, tricaine at higher concentrations also blocks muscle action potentials, probably via effects on Na_v_ channels. Lastly, nifedipine-sensistive L-type voltage-gated calcium channels also act in the nervous system but appear to have little direct action within zebrafish muscle to promote contraction.

Movements in skeletal muscle can be elicited either by neurons (natural movement) or by external electrical stimulation (evoked movement, EES), in which muscle plasma membrane depolarization caused by the applied voltage triggers an action potential. In the current study, we show that EES can initiate contraction in mutants that lack the acetylcholine receptor delta subunits and therefore cannot receive natural input from motorneurons [41]. EES as used here triggers brief sub-tetanic contractions; ramping the voltage beyond 20 V caused more forceful movements (unpublished observations). EES will enable future studies of the impact of muscle electrical activity on gene expression and growth.

Amino amide anaesthetics are known to block Na_v_ channels and thus block the conduction of action potentials in excitable cells [18]. Tricaine is the most commonly used anaesthetic agent for zebrafish. Tricaine is structurally similar to the anaesthetic agents procaine, bupivacaine and lidocaine. These anaesthetics also bind reversibly to Na_v_ channels, blocking Na^+^ movement through the channels, but can also affect other channels [38]. We were able to evoke muscle contraction in fish incubated in tricaine. The magnitude of contraction was similar to that evoked in mutants lacking transmission at the neuromuscular junction. We interpret these observations to show that tricaine does not prevent action potentials in skeletal muscle fibres, whereas action potentials in nerve cells are effectively prevented, leading to general anaesthesia. This finding is important for considerations of animal husbandry; although fish appear to detect and avoid tricaine [23], [28], our data show that tricaine works preferentially at the neural, rather than muscular, level of control of movement. Although questions remain on the most appropriate agents for fish anaesthesia [22], our study shows that tricaine at standard concentrations does not act as a paralytic neuromuscular blocking agent in zebrafish.

Of the known Na_v_ channel alpha subunit genes, only *scn*4*aa* and *scn*4*ab* are expressed in zebrafish skeletal muscle around 3 dpf [30]-[32]. It seems that channels made with these subunits (Na_v_1.4) may be less sensitive to tricaine than those made with classical neural homologues, such as *scn1a* (Na_v_1.1) and *scn8a* (Na_v_1.6) subunits. The Na_v_1.5 channel alpha subunit gene (*scn12ab* in zebrafish) appears to be expressed transiently in early developing slow muscle fibres, but may become restricted to cardiac muscle later [30], [31]. The murine Na_v_1.5 channel is up-regulated in skeletal muscle on denervation [48]. It is therefore possible that the effectiveness of tricaine in inhibiting evoked movement in *fixe* mutants reflects up-regulation of similar channels sensitive to tricaine in inactive somitic muscle. Indeed, mammalian Na_v_1.5 appears several fold more sensitive than Na_v_1.4 to lidocaine [29]. However, the ineffectiveness of tricaine to silence embryonic zebrafish heart demonstrates that the major cardiac Na_v_ channel expressed at early stages (*scn12aa* [31]) is not particularly tricaine sensitive. Interestingly, the heart becomes more sensitive to tricaine as the fish matures, perhaps correlating with increasing predominance of expression of *scn12ab* in the myocardium [27], [49]. Electrophysiological studies of isolated zebrafish channels are required to resolve these issues.

Higher concentrations of tricaine largely inhibit evoked muscle contraction, suggesting either that muscle action potentials can be prevented by this drug or that tricaine may affect channels involved in excitation-contraction coupling. The simplest explanation appears to be that the Na_v_1.4 channels are inhibited by high dose tricaine. Alternatively, Ca_v_ channels contain alpha subunits with structural similarity to Na_v_ channels [2], [12]. As developing muscle has been reported to have Ca_v_ channels capable of supporting a Ca^++^-based action potential [12], these could be targets of high dose tricaine. The *cacna1sa* channel thought to be a muscle-specific subunit of the DHPR is expressed in zebrafish somitic muscle [40]. So high dose tricaine might block excitation-contraction coupling directly.

We addressed the idea of Ca^++^-based action potentials directly, but failed to find evidence in favour of this possibility. Specialised L-type Ca_v_ channels can be blocked by nifedipine [50]. We found that nifedipine inhibits CNS function in zebrafish larvae, leading to reduced movement. However, nifedipine had no effect on the residual motility present in tricaine-treated larvae, indicating that neither evoked action potentials, nor the DHPR, were blocked by this drug. These findings argue that L-type Ca_v_ channels are not responsible for the resistance of zebrafish muscle to normal doses of tricaine.

## Supporting Information

Movie S1 **Movement of wild type (wt),** ***fixe*** **mutants (** ***chrnd^sb13/sb13^*** **) or** ***fixe*** **sibling (** ***chrnd^sb13/+^ or chrnd^+/+^*** **) fish in response to EES.** Fish were treated with the water control or tricaine at standard or fivefold dose, as indicated before each clip. A pulse train of 0.1 s duration was applied every 5 seconds. Movie is sped up about 2.5-fold for ease of viewing. Note increased movement in response to the first pulse, possibly reflecting the need for channel activation for tricaine to bind to Na_v_ channels. Heartbeat shows all fish are alive. In ‘*fixe* + 5X tricaine' clip, heartbeat shows fish are alive but fail to respond to stimulation.
(MP4)
